# Selegiline acts as neuroprotective agent against methamphetamine-prompted mood and cognitive related behavior and neurotoxicity in rats: Involvement of CREB/BDNF and Akt/GSK3 signal pathways

**DOI:** 10.22038/ijbms.2020.38827.9221

**Published:** 2020-05

**Authors:** Saba Feizipour, Sarvenaz Sobhani, Shafagh Mehrafza, Mina Gholami, Majid Motaghinejad, Manijeh Motevalian, Sepideh Safari, Reza Davoudizadeh

**Affiliations:** 1 Department of Pharmaceutical Chemistry, Faculty of Pharmaceutical Chemistry, Pharmaceutical Sciences Branch, Islamic Azad University (IUAPS), Tehran, Iran; 2 Razi Drug Research Center, Iran University of Medical Sciences, Tehran, Iran; 3 Department of Medicinal Chemistry, Faculty of Pharmacy, Tehran University of Medical Sciences, Tehran, Iran; 4 Department of Medicine, Qom branch, Islamic Azad University, Iran

**Keywords:** Anxiety, Depression, Methamphetamine, Neurotoxicity, Selegiline

## Abstract

**Objective(s):**

Present study investigated the neuroprotective effects of selegiline and the molecular mechanisms involved in methamphetamine-induced neurotoxicity.

**Materials and Methods:**

Male wistar rats were randomly divided into six groups (10 rats in each group). Group 1 and group 2 received normal saline and methamphetamine (10 mg/kg), respectively. Groups 3, 4, 5 and 6 were treated simultaneously with methamphetamine and selegiline. From day 22 to day 28, forced swim test, elevated plus maze, and open field test were conducted to assess mood (anxiety and depression) levels, and from day 17 to day 21, Morris Water Maze was conducted for cognition assessment. On day 29, hippocampus of the animals were isolated and evaluated by ELISA method for oxidative, antioxidant, and inflammatory factors and expression levels of active (total) and inactive (phosphorylated) forms of cyclic AMP response element binding protein (CREB), brain-derived neurotrophic factor (BDNF), Akt (Protein Kinase B) and glycogen synthase kinase 3 (GSK3) proteins.

**Results:**

Selegiline reduced behavioral impacts caused by methamphetamine in all doses. Methamphetamine administration may improve malondialdehyde, tumor necrosis factor-alpha, interleukin-1 beta and GSK3 (both forms). Moreover, methamphetamine reduced the activity of superoxide dismutase, glutathione peroxidase, glutathione reductase, amount of BDNF, CREB and Akt (both forms).

**Conclusion:**

Current research showed that selegiline can protect the brain from methamphetamine-prompted neurodegeneration, and this could be intervened by CREB -BDNF or Akt-GSK3 signaling pathways.

## Introduction

Methamphetamine that is prescribed for attention deficiency hyperactivity disorder in children stimulates the central nervous system (1). Continuing impacts of methamphetamine abuse and negative neurochemical and behavioral impacts are still undefined (2). This agent is comparable in functional and pharmacological features to cocaine (3-5). Because of these similarities, methamphetamine has an incredibly elevated potential for abuse and addiction (3-5). 

Various previous studies have shown that extended use and abrupt cessation of methamphetamine may trigger some neurobehavioral disturbances, such as anxiety and depression, which are considered as the major symptoms of methamphetamine withdrawal (3, 4, 6). Researches indicate that the decrease in dopamine and norepinephrine may be responsible for these behaviors over the long term use of methamphetamine and down expression of amine-associated receptors (7-9). In addition, some prior studies have shown that misuse of methamphetamine can interfere with mitochondrial function leading to oxidative stress, inflammation and neurodegeneration in various brain regions (10, 11). Selegiline is an irreversible selective monoamine oxidase-B (MAO-B) inhibitor, but it also inhibits MAO-A in higher doses. This agent is prescribed for Parkinson’s disease therapy, depression and dementia in the early stages (12). Previous studies have found that selegiline may be a useful replacement for anxiolytic and antidepressant impacts in circumstances where both depression and anxiety occur, as with amphetamine and other psychostimulants (13, 14). There are numerous studies on the impact of this agent on the incidence of neurotoxicity due to its anti-apoptotic and anti-oxidant effects (15-17). It has also been shown that selegiline can be effective in the treatment of alcohol, morphine and other drug addiction (18, 19). 

A significant percentage of current research have shown that dopamine and a number of dopaminergic receptor agonists have anxiolytic and antidepressant impacts; these results suggest that a dopaminergic agent such as selegiline may be efficient against methamphetamine-induced anxiety and depression, but its putative mechanism remained imprecise (20, 21). On the other side, cyclic AMP response element binding protein (CREB) functions as a critical transcription factor in neurogenesis and brain regeneration (22-25). Phosphorylated form of CREB (active form) is activated by different protein kinase, which phosphorylates this specific protein and converts CREB into its active form (26-32). CREB, by acting on DNA, stimulates brain-derived neurotrophic factor (BDNF) protein production, which is crucial for neurogenesis and growth. On the other hand, phosphatidylinositol 3-kinase (PI3 K) may activate Akt (Protein Kinase B) protein, which leads to inhibition of glycogen synthase kinase 3 (GSK3), involved in neurodegeneration, and inhibits GSK3 neurodegenerative effects in cells (33). The Akt/GSK3 signaling pathway also plays a critical role in cognitive exercise (33). Due to the significance of the role of P-CREB / BDNF and Akt / GSK3 signaling paths in inhibiting neurodegeneration and modulation of cognition, the current research was considered to evaluate the role of these pathways in the provision of neuroprotective features of selegiline against methamphetamine-prompted neurotoxicity and neurobehavioral complications. This can also lead to a better understanding of the effects of amphetamines and the procedures involved.

## Materials and Methods

***Animals***

Sixty male adult Wistar rats (with average weighing 270 g) were purchased from an experimental research center at the IUMS in Iran. They have been placed in standard situation with free access to food and water. The room temperature was kept at 22±0.5 ^°^C with a light/dark cycle of 12 hr. The investigational procedure of this research was obtained form medical doctorate thesis and all procedure was authorized by research committee in Department of medicine at Qom branch of Islamic Azad University (Research Protocol and ethical code number is 121914).

***Experimental development***

- All animals were randomly distributed into six groups (10 rats per group).
- Group 1 (control group) was administered for 21 days with normal saline (0.2 ml / rat, IP once daily).
- Group 2 (methamphetamine-treated group) had methamphetamine for 21 days (Sigma-Aldrich, USA) (10 mg/kg, IP once a day).
- Methamphetamine (10 mg/kg, IP once a day) and selegiline (Sigma-Aldrich, USA) 5, 10, 15 and 20 mg/kg doses were given concurrently during 21 days in groups 3, 4, 5 and 6.

During day 22-28, some standard behavioral approaches such as Forced Swim Test (FST), Elevated Plus Maze (EPM) and Open Field Test (OFT) were used to determine mood-related activity (anxiety and depression) levels in experimental animals. Furthermore, Morris water maze (MWM) protocol was introduced to evaluate spatial memory and learning in animal treatment groups between the 17^th^ and 21^st^ days. On day 29, all animals were anesthetized by administering thiopental (50 mg/kg), removing brain tissue and separating their hippocampus from each rat according to previous study guides (34, 35). It has to be noted that the hippocampus in right hemisphere has been used to assess biomarkers for oxidative stress and inflammation, and hippocampus in left hemisphere has been used to assess CREB, P-CREB, BDNF, Akt, P-Akt, GSK and P-GSK expression of proteins.

***Behavioral studies***

*Open Field Test*

The open-field device was used for evaluation of anxiety and motor activity disorder, and this test was performed according to standard protocol reported in previous studies (36, 37). According to this protocol, 4 standard behaviors were evaluated in this test.

1. Line crossing (ambulation) distance: Distance of the rat passing through the grid lines.

2. Center Square Entries: Frequency of that the rat crossed one of the red lines with all four paws in the main square.

3. Center Square Duration: The length of time the rat spent on the main square.

4. Rearing number: The frequency with which the rat shows a strange behavior (36, 37).

*Forced Swim Test*

This is the assessment of behavior that is deployed in experimental designs to evaluate depression-like behaviors. This test was performed according to standard protocol described by previous studies. Swimming exercise demonstrated non-depressive behavior, while immobility demonstrated depressive behavior (37-39).

*Elevated plus maze*

EPM is a commonly deployed test for evaluation of anxiety-related behavior in rodents. Protocol of this test was performed according to standard protocol reported by previous studies. The tendency to be in the closed arms over the open arms was representative of anxiety-like actions, while tendency to be in the open arms over the closed arms was representative of open arm over the closed arms was Representative of anti anxiety action (39, 40).

*Morris water maze Task*

A unit of the MWM has been used for both learning and memory behavior, and the protocol of learning and memory assessment was performed according to previous standard protocols (41, 42). 

During the learning process, three variables were evaluated.

1. Time to escape latency defined by time to discover the hidden platform.

2. Traveled distance that has been verified by the time every animal has spent entering and locating the concealed platform.

3. Speed of the animal during the discovery of a concealed stand.

Through the evaluation of memory, the platform was removed on the fifth day (sample day), and the animals were randomly terrified by water from one of the above-mentioned directions (near East), and the proportion of animal presence in the target quarter (South East corner) was reported and measured (41, 42).

***Molecular study***

*Mitochondrial preparations*

Thiopental sodium (50 mg/kg, IP) was used to anesthetize animals and the hippocampus was removed from each rat. The isolated tissues were prepared according to standard protocol of mitochondrial isolation and were tested to assess oxidative stress and inflammatory markers (35, 43-45). 

*Measurement of oxidative stress biomarker*

Determination of oxidative stress parameters, Malondialdehyde (MDA) level and also activity of antioxidant enzymes such as manganese superoxide dismutase (MnSOD), glutathione peroxidase (GPx) and glutathione reductase (GR) was performed according to previous standard protocols (44, 46-51). 

*Measurement of expression of protein*

Concentrations (protein levels) of BDNF, CREB (complete and phosphorylated), Akt1 (Protein Kinase B) (total and phosphorylated), GSK3 (total and phosphorylated), interleukin-1 beta (IL-1β) and tumor necrosis factor-alpha (TNF-α) in hippocampal tissue cell lysate were evaluated using an ELISA kit, which is commercially available (Genzyme Diagnostics, Cambridge, U.S.A) with special protocol and standard procedure (52-55). Results of BDNF, CREB (complete and phosphorylated), Akt1 (total and phosphorylated) and GSK3 (total and phosphorylated) were stated as pg/ml in tissue suspension of the hippocampus, while TNF-α and IL-1β were reported as ng/ml (52-55).

***Analysis of statistics***

The information collected was evaluated by GraphPad PRISM v.6 Software and averaged in each test group and stated as a mean±standard error (SEM). ANOVA was also used in the MWM experiment to assess behavior over four days of practice and its differences were evaluated on a daily basis. The distinctions between control and therapy groups were then assessed by ANOVA. To assess the severity of behaviors, variations between means in groups were compared using the Tukey’s* post-test* at a substantial rate of (*P*<0.001) or (*P*<0.05).

## Results

***OFT behavior in experimental group***

As shown in Table 1, methamphetamine (10 mg/kg)-treated animals have lower rates of central square entries, less time spent in the core area, and higher frequency of OFT rearing and ambulation distance (*P*<0.05)(Table 1). Selegiline inhibited this dose-dependent effect of methamphetamine and improved central square entry rate, core time, OFT breeding frequency and ambulation period in methamphetamine-treated groups. Compared to methamphetamine (10 mg/kg), this distinction was statistically significant (*P*<0.05) (Table 1). Selegiline also improved mentioned OFT behaviors in animals treated with methamphetamine (10 mg/kg) at the highest dose (20 mg/kg). This improve was remarkably significant relative to the methamphetamine group (10 mg/kg) alone (*P*<0.05) (Table 1). Testament of methamphetamine-dependent animals with high doses of selegiline (20 mg/kg) results in remarkable differences in the rate of OFT parameters such as rearing, central square entries and time spent in the central area compared to animals treated with low doses of selegiline (5 mg/kg) (*P*<0.05) (Table 1).

***FST behavior in in experimental group***

**Figure 1:**
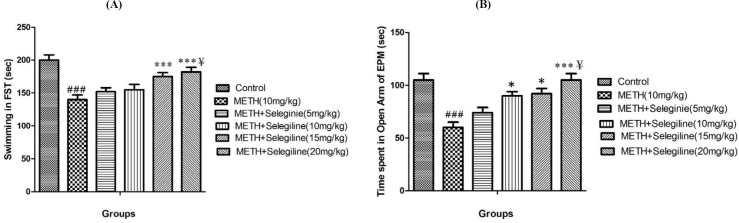
Displays swimming time (sec) in forced swim test (FST)

Rats in the treated group of methamphetamine (10 mg/kg) indicated less swimming time compared to the control group in FST (*P*<0.001) (Figure 1A). Selegiline inhibited the impact of methamphetamine at all doses and decreased the swimming time of experimental animals. Nonetheless, selegiline at doses of 15 and 20 mg/kg significantly improved swimming time compared to the group which received only methamphetamine (10 mg/kg) (*P*<0.001)(Figure 1A). Significant differences in swimming time were observed in methamphetamine-dependent animals treated with high doses of selegiline (20 mg/kg) relative to animals treated with low doses of selegiline (5 mg/kg) (*P*<0.05) (Figure 1A).

***EPM behavior in in experimental group***

Animal of control group stayed more time in the open arms of EPM in comparison with the group treated with 10 mg/kg of methamphetamine (*P*<0.001) (Figure 1B). This study showed that selegiline treatment with doses of 10, 15 and 20 mg/kg significantly reduced the existence of animals in the open arms of EPM (*P*<0.05 for 10 mg/kg selegiline and* P*<0.001 for 15 and 20 mg/kg selegiline) relative to only methamphetamine (10 mg/kg)-treated group (Figure 1B). Results showed significant time gaps for methamphetamine-dependent animals treated with high doses of selegiline (20 mg/kg) in the open arms of EPM relative to animals treated with 5 mg/kg of selegiline (*P*<0.05) (Figure 1B).

***Evaluation of learning parameters (escape latency and traveled distance) in MWM***

**Figure 2:**
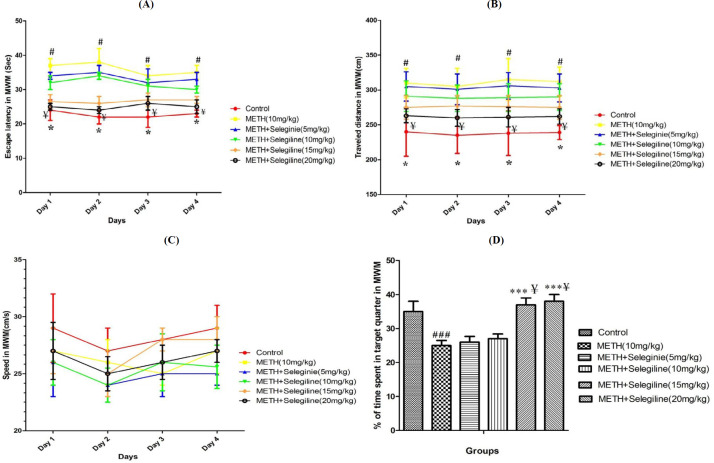
Displays mean of escape latency (A), traveled distance (B), swimming speed (C) during four days of training and percentage of time spent in target quarter in probe trial(D) in Morris Water Maze (MWM) in rats under treatment in control group and groups treated by methamphetamine (10 mg/kg) and selegiline (5, 10, 15 and 20 mg/kg) in combination with methamphetamine. All data are expressed as Mean±SEM (N=10)

Escape latency and traveled distance for the group treated with methamphetamine at a dosage of 10 mg/kg during four days of training in the MWM was statistically significant compared to the control group, and this behavior was different during all four days of training (learning) (*P*<0.05) (Figure 2A and B). While selegiline inhibited increases in escape latency caused by methamphetamine in all doses and traveled distance, and these differences were statistically significant at the 15 and 20 mg/kg doses of selegiline compared to only methamphetamine (10 mg/kg)-treated group (*P*<0.05) (Figure 2A and B). High doses of selegiline (20 mg/kg), in methamphetamine-dependent animals, significantly increased latency and traveled distance compared to animals under therapy with small doses of selegiline (5 mg/kg) (*P*<0.05) (Figure 2A and B).

***Evaluation of swimming speed in MWM***

During training trials, the mean swimming speeds were not changed in any of the animal groups, indicating that exposure to methamphetamine (10 mg/kg) alone or in addition to selegiline (5, 10, 15 and 20 mg/kg) did not lead to motor disruption in test animals (Figure 2C).

***Percentage analysis in the target quarter in the MWM probe trial***

Findings showed that there is a significant reduction in the proportion of animal presence in the target quarter of methamphetamine (10 mg/kg)-treated group compared to the control group (*P*<0.001) (Figure 2D). Selegiline with 15 and 20 mg/kg doses can also reduce this impact of methamphetamine, which was statistically relevant relative to methamphetamine alone (10 mg/kg)-treated group (*P*<0.001) (Figure 2D). Treatment with high doses of selegiline (20 mg/kg) in methamphetamine-dependent animals resulted in significant differences in animal presence (as percentage) in the target quarter relative to animals treated with low doses of selegiline (5 mg/kg) (*P*<0.05) (Figure 2D).

***Evaluation of oxidative stress changes***

Methamphetamine administration significantly increased MDA (lipid peroxidation biomarker) levels and also decreased SOD, GPx and GR activities compared to the control group (*P*<0.05) (Table 2). By comparison, varying doses of selegiline (5, 10, 15 and 20 mg/kg) decreased methamphetamine-induced levels of MDA and reduced methamphetamine-prompted reductions in SOD, GPx and GR behaviors relative to groups treated with methamphetamine (*P*<0.05) (Table 2). In methamphetamine-dependent animals treated with 10 and 20 mg/kg of selegiline, there were substantial differences in levels of MDA and SOD, GPx and GR activity relative to treatment-dependent animals with small doses of selegiline (5 mg/kg) (*P*<0.05)(Table 2).

***Evaluation of inflammation alteration***

The animals in methamphetamine-treated groups showed significant rises in IL-1β and TNF-α levels in comparison with the control group (*P*<0.05) (Table 2). While, high selegiline doses (15 and 20 mg/kg) inhibited methamphetamine-induced rise in biomarkers of inflammation relative to methamphetamine-only treatment (*P*<0.05) groups (Table 2). The increase in IL-1β and TNF-α concentrations in methamphetamine-dependent animals treated with 10 and 20 mg/kg selegiline was significant relative to the 5 mg/kg selegiline-treated methamphetamine-dependent animal (*P*<0.05) (Table 2).

***Results of selegiline effects on methamphetamine-prompted alterations in CREB, P-CREB and BDNF protein expression***

**Figure 3:**
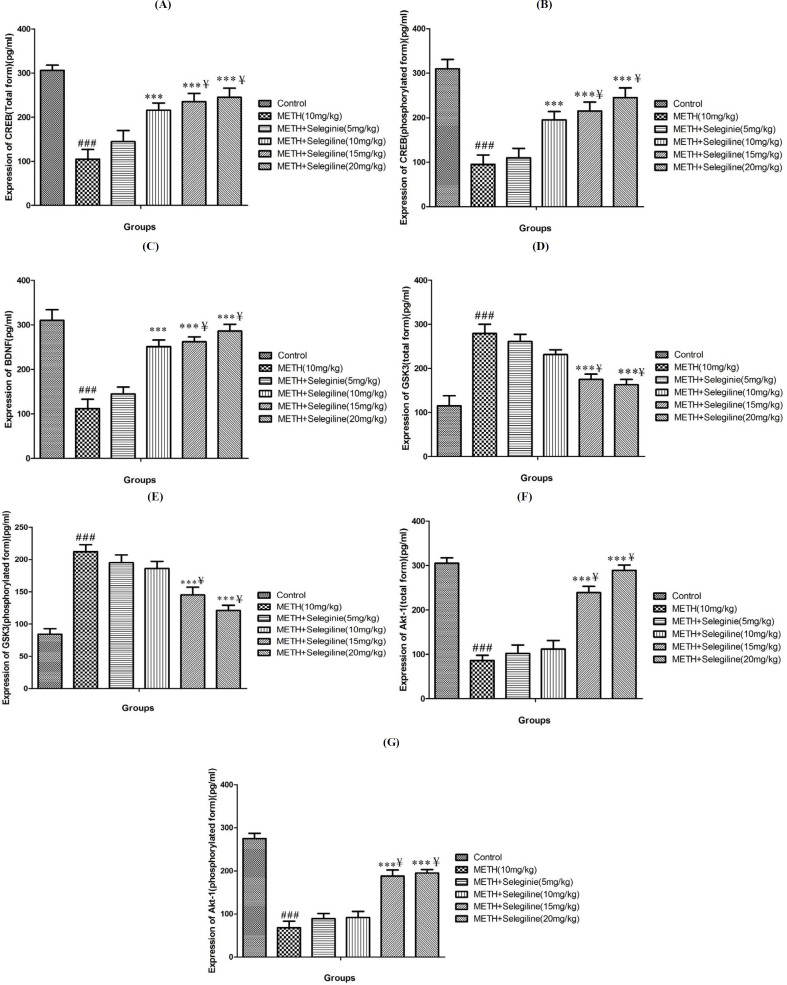
Shows alterations in expression/level (ELISA) of CREB (total form)(A), CREB (phosphorylated form) (B), BDNF (C), GSK3(total form)(D), GSK3 (phosphorylated form)(E), Akt (total form) (F) and Akt (phosphorylated form)(G) in hippocampus of rats under treatments in control group and group under treatment with 10 mg/kg of methamphetamine and groups under treatment by methamphetamine in combination with selegiline (5, 10, 15 and 20 mg/kg). All data are expressed as Mean±SEM (N=10)

Methamphetamine (10 mg/kg) therapy significantly decreased CREB (total and phosphorylated) and BDNF relative protein levels (expression) in rat hippocampus compared to control group (*P*<0.001) (Figure 3A, B and C). Selegiline (10, 15 and 20 mg/kg) levels, on the other hand, significantly increased the protein levels (expression) of CREB (both form) and BDNF in methamphetamine-treated animals relative to the groups treated with methamphetamine alone group (*P*<0.001) (Figure 3A, B and C). Findings showed substantial differences in the extent of CREB protein (expression) (both form) and BDNF from methamphetamine-dependent animals treated with 10 and 20 mg/kg selegiline compared to animals treated with small doses of selegiline (5 mg/kg) (Figures 3A, B and C) (*P*<0.05).

***Results of selegiline effects on methamphetamine-prompted alterations in Akt and GSK3 proteins expression***

Methamphetamine (10 mg/kg) therapy significantly decreased Akt (total and phosphorylated) relative protein content (expression) and increased relative extent of GSK3 protein (expression) (total and phosphorylated) in rat hippocampus compared to the control group (*P*<0.001) (Figure 3D, E, F and G). On the other hand, selegiline at elevated doses (15 and 20 mg/kg) considerably enhanced the protein level (expression) of Akt (total and phosphorylated) and reduced the level (expression) of GSK3 (total and phosphorylated) in animals treated with methamphetamine relative to the methamphetamine only-treated group (*P*<0.001) (Figure 3D, E, F and G). Results in methamphetamine-dependent animals treated with 10 and 20 mg/kg selegiline showed significant differences in protein level of (expression) Akt (both forms) and reduced level (expression) of GSK3 (both form) compared to methamphetamine-dependent animals treated with small doses of selegiline (5 mg/kg) (*P*<0.05) (Figure 3D, E, F and G). 

## Discussion

Present research indicated that selegiline at various doses can alter methamphetamine-prompted neurotoxicity and the sequels of neurobehavior such as mood disorder (anxiety and depression) and cognitive defect. According to our results, there is a chance that selegiline could inhibit behavioral and molecular alterations caused by methamphetamine administration by modulating the CREB/BDNF and Akt/GSK3 signaling paths. Results of the study showed that methamphetamine (10 mg/kg) reduced entry and time spent in the central square of OFT and also led to distance ambulation and rearing disturbances. Data showed that the amount of methamphetamine used in this research could lead to disruption in motor operation. 

On the other side, our findings showed that in all of the above doses, selegiline lowered the OFT behavioral parameters in rat treated with methamphetamine. Our findings are similar to prior research, which showed that selegiline can change depression in rats and can modulate disruption of motor activity caused by some drug abuse (56, 57). 

Numerous fundamental studies have shown that certain brain amines such as norepinephrine and dopamine play a critical role in the leadership of motor exercise (58, 59). Conferring to this fundamental notion, it can be interpreted that methamphetamine can persuade behavioral illnesses in OFT through disturbance in this form of amine. It can therefore be suggested that selegeline, possibly through modulation of norepinephrine and dopamine release, disrupted by methamphetamine, can prevent adverse effects and normalize OFT in rat treated with methamphetamine (13, 60, 61). The results of current research have shown that Methamphetamine 10 mg/kg can decrease swimming time in FST and time of spent in open arms (sec) in EPM, while selegiline (10, 15 and 20 mg/kg) may reduce this form of depression (immobility sign) in FST and anxiety (less time spent in open arms). Selegiline (15 and 20 mg/kg) can also increase swimming time in FST and the quantity of time spent in the open arm. Long-term abuse of methamphetamine may decrease amine-based neurotransmitters engaged in anxiety and depressive behavior, and it appears that these kinds of depletion are accountable for depressive animal behavior under methamphetamine therapy in our research (62). Selegiline, owing to its antidepressant impact, can enhance dopamine in brain synapses and regulate depressive conduct in rats and can compensate for methamphetamine-induced dopamine depletion (13, 63). Conferring our information and previous outcomes, it is verified that even sub-therapeutic doses of selegiline can be efficient in FST and EPM and can enhance mood (13). 

According to our research, extended methamphetamine administration at the mentioned doses can cause enhancement of latency escaped and distance traveled throughout four days of MWM. Practice and these behaviors were statistically important in all four days. With regard to learning time and information, it may be suggested that methamphetamine at the aforementioned dose may reduce learning activity and the proportion of probe day presence in the target quarter of MWM. Our finding confirms the outcomes of prior research that indicated that chronic methamphetamine administration can disrupt spatial cognitive ability by depletion of dopamine, serotonin and adrenaline in rats (10, 64). On the other side, our data showed that selegiline could influence changes in learning and spatial memory induced by methamphetamine at each of the listed doses, particularly in high doses (15 and 20 mg/kg). In line with our information, many past trials have shown that selegiline and other MAO-B inhibitors have an important beneficial effect in enhancement of cognition (61, 65). According to this research, selegiline can behave as an efficient antidepressant, anxiolytic and also cognitive enhancer, and can be used against methamphetamine and other drug abuse-induced behavioral disturbances. With regard to the optimal dose of selegiline in the management of behavioral parameters, our data showed that all the mentioned doses of selegiline could modulate OFT behaviors. However, high doses (15 and 20 mg/kg) of selegiline could prevent methamphetamine-induced mood disorder and impairment of cognition in FST, EPM, and MWM. This information is consistent with previous outcomes showing that selegiline in sub-therapeutic doses can change motor activity in experimental scales (66, 67). While, greater doses of selegiline are efficient for enhancing cognition and mood-related behavior (13). Consistent with our behavioral outcomes, our molecular findings have shown that methamphetamine (10 mg/kg) can change the condition of oxidative stress and neuro-inflammation. The current research stated that methamphetamine may reduce SOD, GPx, and GR activities, while improving the amount of MDA as a marker of lipid peroxidation, as well as TNF-α and IL-1β in rat hippocampus. Some parts of methamphetamine toxic impacts can be asserted to be modulated by inhibition of SOD, GPx and GR actions, depletion of antioxidant enzymes, induction of lipid peroxidation, induction of neuro-inflammation, and disturbance of mitochondrial function (62, 63). Our data are compatible with earlier research that stated that chronic methamphetamine administration induced mitochondrial dysfunction and modification in respiratory chain proteins, as well as activation of neuro-inflammation in rodent brain cells (68, 69). Nevertheless, the precise mechanism of methamphetamine action in this respect remains uncertain (69). The results of the present research are also comparable to prior findings on methamphetamine-induced lipid peroxidation and neuro-inflammation in brain cells (10, 70). The results of recent studies have shown that methamphetamine consumption prevents antioxidant activity and stimulates neuro-inflammation in various cells, and these impacts trigger methamphetamine-induced degenerative impacts on body cells such as brain, liver, heart, and other cells (70). In the present research, selegiline therapy (in all doses used) was discovered to be efficient in reversing this methamphetamine-induced rise in MDA, TNF-α and IL-1β and in reversing SOD, GPx and GR changes in hippocampal tissue (71, 72). Selegiline may be suggested to modulate methamphetamine-induced neurodegeneration and neurotoxicity by activation of mitochondrial biogenesis, antioxidant enzymes or inhibition of inflammation and lipid peroxidation (73). Numerous trials have endorsed the function of selegiline in activating antioxidant defense, inhibiting inflammation and also increasing the activity of antioxidant enzymes (72). Many researches have shown that selegiline and other comparable agents can act as mitochondrial enzyme activators and these properties involve the effects of selegiline preventive effects on neurotoxicity caused by morphine and amphetamine (71, 72, 74). We assessed the molecular basis and the likely signaling paths involved in this action in order to identify the mechanism of neuroprotective behavior of selegiline toward methamphetamine-induced behavioral and molecular effects. Thus, we assessed the signaling pathways for P-CREB / BDNF and Akt / GSK3. According to our data, methamphetamine (10 mg/kg) can reduce the expression levels of CREB and Akt proteins in complete and phosphorylated forms and decrease BDNF. Our findings also showed that methamphetamine can increase the expression level of GSK3 protein in complete and phosphorylated forms. These findings are consistent with prior work showing that methamphetamine type stimulant can inhibit P-CREB and Akt phosphorylation in brain cells and inhibit BDNF production and enhance GSK3 phosphorylation leading to neurodegeneration (64, 75, 76). Our data also showed that selegiline can inhibit methamphetamine-induced decline in the expression level of CREB protein in complete and phosphorylated form, thus increasing the output of BDNF in rats treated with methamphetamine. These selegiline impacts were shown at 10, 15, and 20 mg/kg doses. Our data also shows that selegiline, particularly at greater doses (15 and 20 mg/kg), can prevent methamphetamine-prompted rises in GSK3 and reduction in protein expression level of Akt (33). Based on this finding, it can be discussed that selegiline can likely handle and reverse methamphetamine-induced neurodegeneration through activation of P-CERB formation, induction of BDNF manufacturing, and modulation of GSK3 (inhibition) and Akt (activation)(77, 78). With regard to the role of small and high doses of selegiline in the management of molecular parameters, our finding showed that all the aforementioned doses of selegiline could modulate oxidative stress and inflammatory biomarkers in accordance with previous information showing their neuroprotective effects at low doses (71). Selegiline could modulate methamphetamine-induced changes in P-CREB / BDNF at doses of 10, 15 and 20 mg/kg and alter Akt / P-GSK3 proteins at doses of 15 and 20 mg/kg to verify its role in signaling pathway modifications at mild to elevated doses (79). Conferring to the current results, selegiline and other comparable agents may behave via P-CREB / BDNF or P-Akt / P-GSK3 paths, and activate neuroprotection. These novel findings offer new insights into the molecular basis of protective impacts of selegiline and also toxic impacts of methamphetamine in hippocampal cells.

**Table 1:** The effects of various doses of selegiline on open field exploratory and anxiety like behavior in rats treated with 10 mg/kg of methamphetamine

**Group**	**Ambulation distance** **(cm)**	**C** **entral square entries** **(number)**	**Time spent in central ** **square(sec)**	**Number of rearing**
**Control (NS)**	325±16	27±3	160±8	12±2
**METH** ** (10mg/kg)**	210±17^a^	14±2.5^a^	109±7^ a^	5±1^ a^
**METH(1** **0mg/kg)** ** +Selegiline (5** **mg/kg)**	260±20^ b^	16±2	130±9^ b^	6±1
**METH(1** **0mg/kg)** ** +Selegiline (10** **mg/kg)**	270±18^ b^	17±1.8^b^	135±8^ b^	9±1
**METH(1** **0mg/kg)** ** +Selegiline (15** **mg/kg)**	275±22^ b^	23±2^ b &c^	149±9^ b &c^	9±2.5^ b &c^
**METH(1** **0mg/kg)** ** +Selegiline (20** **mg/kg)**	300±18^ b^	24±1.5^ b &c^	151±8^ b &c^	10±1.5^ b &c^

^a^ represents statistical significance vs. control group (*P<*0.05)

^b^ represents statistical significance vs. methamphetamine group (10 mg/kg) (*P<*0.05)

^c^ represents statistical significance vs. 10 mg/kg of methamphetamine in combination with 5 mg/kg of selegiline (*P<*0.05)

All data are expressed as mean±SEM (N=10)

METH: methamphetamine; NS: Normal Saline

A: duration of time spent in open arms (sec) in elevated plus maze (EPM), B: In control group, and groups treated with 10 mg/kg of methamphetamine and selegiline (5, 10, 15 and 20 mg/kg) in combination with methamphetamine. All data are expressed as mean±SEM=10)

### *P<* 0.001 vs control group, * *P<*0.05 vs. 10 mg/kg of methamphetamine, *** *P<*0.001 vs 10 mg/kg of methamphetamine, ¥ represents statistical significance with *P<*0.05 vs 10 mg/kg of methamphetamine in combination with 5 mg/kg of selegiline

METH: methamphetamine

* *P<*0.05 vs group under treatment with methamphetamine. *** *P<*0.001 vs 10 mg/kg of methamphetamine

# *P<*0.05 vs groups under treatment with 15 and 20 mg/kg of selegiline. ### *P<*0.001 vs control group

¥ represents statistical significance with *P<*0.05 vs 10 mg/kg of methamphetamine in combination with 5 mg/kg of selegiline.

METH: methamphetamine

**Table 2:** The effects of various doses of selegiline on alterations of oxidative stress and inflammatory biomarkers in mitochondria of rats treated with methamphetamine (10 mg/kg/day)

**Group**	**MDA** **nmol/mg** ** of** ** protein**	**SOD** **U/ml/mg protein**	**GPx** **U/ml/mg protein**	**GR** **U/ml/mg protein**	**TNF-α** **ng/ml**	**IL-1β** **ng/ml**
**Control (NS)**	7.2±0.8	69.3±5.2	75.1±3.9	55.1±6.3	59.4±7.3	48.2±6.1
**METH** **(10mg/kg)**	25.4±1.1^a^	36.5±5.5^a^	36.2±6.4^a^	18.1±4.6^a^	106.5±9.9^ a^	109.2±9.1^a^
**METH(1** **0mg/kg)** ** +Selegiline (5** **mg/kg)**	15±1.5^ b^	49.4±6.3^b^	48.2±8.3^ b^	23.4±6.6^ b^	99.5±6.1	95.2±6.1
**METH(1** **0mg/kg)** ** +Selegiline (10** **mg/kg)**	13±3^ b^	45.8±6.1^b^	54.2±8.1^b^	46.3±6.3^ b^	93.1±4.2	92.5±3.1
**METH(1** **0mg/kg)** ** +Selegiline (15** **mg/kg)**	11±0.1^ b &c^	41.6±6.6^b^	67.9±6.3^ b &c^	47.2±5.2^ b &c^	75.6±6.6^ b &c^	83.2±4.2^ b &c^
**METH(1** **0mg/kg)** ** +Selegiline (20** **mg/kg)**	9±0.9^ b &c^	40.3±7.1^b^	71.9±8.9^ b &c^	48.1±6.1^ b &c^	68.1±1.1^ b &c^	71.1±4.3^ b &c^

^a^ represents statistical significance with *P<*0.05 vs. control group

^b^ represents statistical significance with *P<*0.05 vs 10 mg/kg of methamphetamine

^c^ represents statistical significance with *P<*0.05 vs 10 mg/kg of methamphetamine in combination with 5 mg/kg of selegiline

All data are expressed as mean±SEM (N=10)

METH: methamphetamine. NS: Normal Saline. MDA: Malondialdehyde. SOD: Superoxide dismutase. GPx: Glutathione peroxidase. GR: Glutathione reductase. TNF-α: Tumor necrosis factor alpha. IL-1β: Interleukin-1 beta

### *P<*0.001 vs control group. *** *P<*0.001 vs 10 mg/kg of methamphetamine

¥ represents statistical significance with *P<*0.05 vs 10mg/kg of methamphetamine in combination with 5 mg/kg of selegiline

CREB: Cyclic AMP response element binding protein. BDNF: Brain-derived neurotrophic factor. Akt: Protein kinase B. GSK3: Glycogen synthase kinase 3. METH: methamphetamine

## Conclusion

The present research showed that the signaling pathway for P-CREB / BDNF and P-Akt/P-GSK3 could be engaged in selegiline protective impacts against methamphetamine-prompted behavioral and molecular sequels. Although these data give new insights about unknown mechanisms of methamphetamine-prompted neurotoxicity, but further precise molecular and cellular assessment of protective role of selegiline against methamphetamine-prompted sequels seems necessary.
